# Relationship between Non-Energy-Adjusted and Energy-Adjusted Dietary Inflammatory Index and the Healthy Eating Index-2015: an analysis of the National Health and Nutrition Examination Survey (NHANES) 2015–2018

**DOI:** 10.1080/07853890.2023.2236551

**Published:** 2023-07-25

**Authors:** Janie C. DiNatale, Deniz Azarmanesh, James R. Hébert, Michael D. Wirth, Jessica Pearlman, Kristi M. Crowe-White

**Affiliations:** a Department of Human Nutrition, The University of Alabama, Tuscaloosa, AL, USA; b Department of Human Nutrition, Connecting Health Innovations LLC, University of SC, Columbia, SC, USA; c Cancer Prevention and Control Program, University of South Carolina, Columbia, SC, USA; d Department of Epidemiology Biostatistics, Arnold School of Public Health, University of South Carolina, Columbia, SC, USA; e College of Nursing, University of South Carolina, Columbia, SC, USA; f Institute for Social Science Research, University of MA Amherst, Amherst, MA, USA

**Keywords:** National health and nutrition examination survey, healthy eating index, dietary inflammatory index, energy-adjusted dietary inflammatory index, inflammation, dietary patterns, diet quality

## Abstract

**Objectives:**

Acknowledging the association between diet and systemic inflammation, the Dietary Inflammatory Index (DII^®^) and the Energy-Adjusted DII (E-DII^TM^) were developed to categorize diet from anti- to pro-inflammatory. The purpose of this study was to evaluate differences in the relationship between DII and E-DII against the Healthy Eating Index (HEI) to assess the use of energy-adjustment when analyzing the inflammatory potential of the diet.

**Methods:**

This cross-sectional secondary data analysis included 5289 adults participating in the National Health and Nutrition Examination Survey (NHANES) between 2015 and 2018. DII and E-DII scores were calculated and grouped into quartiles. Multivariable linear regression was used to evaluate the association between HEI with DII and E-DII separately, adjusting for age, gender, race/ethnicity, education, family-to-poverty ratio and body mass index. Bootstrap methods were used to estimate the difference between coefficients for E-DII and DII from their respective models.

**Results:**

Results suggest that HEI scores were significantly lower between quartile 2 and quartile 1 of DII scores (Q2 vs. Q1: *β*(SE) = −7.45(0.54), *p* < 0.05) and quartile 3 and quartile 4 against quartile 1 of E-DII scores (Q3 vs. Q1: *β*(SE) = −16.56(0.65), *p* < 0.05 and Q4 vs. Q1: *β*(SE) = −24.93(0.87), *p* < 0.05) in unadjusted models. Similar results were observed in adjusted models (HEI and DII Q3 vs. Q1: *β*(SE) = −10.68(0.82), *p* = 0.049; HEI and E-DII Q2 vs. Q1: *β*(SE) = −9.14(0.64) and Q3 vs. Q1: *β*(SE) = −15.76(0.44) and Q4 vs. Q1: *β*(SE) = −23.77(0.58), *p* < 0.05). Further, 52% of the variance in HEI scores was explained by the E-DII in both adjusted and unadjusted models (*R*^2^ = 0.52). In contrast, 17% of the variance in HEI score is explained by the DII in the unadjusted model (*R*^2^ = 0.17), yet this increased to 26% of the variance in the adjusted model (*R*^2^ = 0.26). The difference between HEI scores for the first versus the fourth quartile of DII scores (−15.64) was significantly larger than the difference between HEI scores for the first versus the fourth quartile of E-DII scores (−25.90; bootstrap estimated 95% CI: 53.41–62.41).

**Conclusions:**

Use of an inflammatory index along with the HEI may provide further understanding into relationships between dietary quality by nutrient and food group consumption on the inflammatory potential of the diet.

## KEY MESSAGES

1. Significant inverse associations exist between HEI and both DII® and E-DII^TM^ scores among a sample of American adults using NHANES 2015–2018 data.
2. HEI scores were the highest in the first E-DII quartile compared to the first DII quartile.
3. Use of an inflammatory index along with the HEI provides further understanding of the associations between diet quality as described by nutrient content and consumption of specific food groups on inflammation and inflammatory-related chronic diseases.

## Introduction

Diet is one of the strongest moderators of inflammation, and unhealthy dietary patterns, such as a traditional Western-style diet, are associated with higher systemic inflammation [1]. In contrast, healthy dietary patterns, such as a Mediterranean diet, are associated with lower systemic inflammation [2,3]. Importantly, systemic inflammation is associated with a higher risk of chronic diseases including cancer and cardiovascular diseases among others [4–6]. Acknowledging these relationships, the Dietary Inflammatory Index (DII^®^) was developed to categorize diet on a continuum from anti-inflammatory to pro-inflammatory [7,8]. The DII was created based on research assessing diet against inflammatory biomarkers and is standardized against a world average dietary intake [7]. For interpretation, higher DII scores represent a more pro-inflammatory diet, whereas lower DII scores represent a more anti-inflammatory diet. While DII scores do not account for energy intake in the calculation, the Energy-Adjusted DII (E-DII^TM^) was created to account for differences in total caloric intake. E-DII scores are based on an intake per 1000 kilocalories consumed. In most studies, the E-DII has been shown to improve the prediction of the inflammatory potential of the diet as compared to non-energy adjusted DII scores [8]. Similar to the DII, higher E-DII scores represent a more pro-inflammatory diet, whereas lower E-DII scores represent a more anti-inflammatory diet.

While the DII and E-DII measure the inflammatory potential of the diet, the Healthy Eating Index (HEI) measures dietary quality by assessing how well a set of foods aligns with key recommendations set forth by the Dietary Guidelines for Americans (DGA) for health and wellness [9]. Originally developed in 1995, the HEI, an energy-adjusted measure of diet quality, has been updated every 5 years [9–11]. In summary, HEI measures dietary quality based on established, *a priori* dietary guidelines.

While the HEI is a metric of dietary quality calculated based on U.S. Dietary Guidelines, the HEI has also been used in international populations. Previous studies demonstrated associations between the HEI and DII in many countries including the United States, Iran, Australia, England and Brazil with higher HEI scores (greater adherence to the DGA) correlated with lower DII scores (more anti-inflammatory diet) in these adult populations [12–14]. Conversely, research assessing the relationship between HEI and the E-DII is limited with only one study investigating this relationship among Iranian adults [15]. In this study, an inverse relationship was reported. While the relationship between HEI with E-DII and HEI with DII separately have been assessed, a comparative assessment of both the DII and E-DII together against a validated national dietary standard is not currently available in the literature [12–15]. Acknowledging the difference in energy adjustment between the two dietary inflammatory indices, investigation of HEI against the DII and E-DII is warranted in order to assess whether the 1000-kilocalorie adjustment leads to better agreement of results [16]. Using the HEI, a standard dietary metric, as a comparator, the potential of a 1000-kilocalorie adjustment while measuring the inflammatory potential of the diet was assessed. Understanding this relationship may assist in proper inflammatory assessment of the diet along with diet quality. Additionally, this analysis may identify an accurate measurement of diet by nutrients (DII/E-DII) to accompany the measurement of diet by food groups (HEI) in research. As such, the purpose of this study was to evaluate the relationship between HEI and both DII and E-DII scores among adults participating in the National Health and Nutrition Examination Survey (NHANES). It was hypothesized that the relationship between the HEI and E-DII would be more closely associated than that of the HEI and DII due to the energy adjustment of the HEI and E-DII. Additionally, it was hypothesized that higher HEI scores (greater adherence to the DGA) would be associated with lower DII and E-DII scores (more anti-inflammatory diet).

## Materials and methods

### Study sample

This cross-sectional secondary data analysis included a sample of adults participating in NHANES between the years 2015–2018. NHANES is a cross-sectional survey of a nationally representative sample of community-dwelling adults and children in the United States that collects demographic, socioeconomic, dietary and health-related information [17]. For purposes of this analysis, participants were included if they were an adult over 20 years old with two valid 24-hour diet recalls. Participant inclusion criteria for age was determined to remain consistent with the adult age cutoff of Dietary Reference Intakes [18]. Pregnant women were excluded due to changes in the overall dietary intake that occur during pregnancy [19]. Ethical approval was not required for this secondary data analysis as the data were previously collected and made publicly available through the Center for Disease Control and Prevention. NHANES was approved by the Research Ethics Review Board of the National Center for Health Statistics (NCHS) and all participants of NHANES gave written informed consent prior to any data being collected.

### Dietary intake

NHANES dietary intake was collected by trained interviewers via computer-assisted software [17]. The average of two 24-hour recalls was used for these analyses [20].

### HEI-2015

The HEI-2015 was calculated using the scoring algorithm from the National Cancer Institute [10]. The HEI-2015 is both density-based and energy-adjusted, with dietary intake assessed per 1000 kilocalories. There are 13 components used for scoring reflecting different food groups and key recommendations by the DGA for adherence to adequacy and moderation components. Nine of these components evaluate adequacy (total fruit, whole fruit, total vegetables, greens and beans, whole grains, dairy, total protein foods, seafood and plant proteins and fatty acids) and four of these components evaluate moderation (refined grains, sodium, added sugars and saturated fat). Adequacy components have a maximum score of five with higher scores translating to higher intake. In contrast, higher scores for moderation components indicate lower intake as lower intakes are more desirable. The scoring components for the HEI-2015 are provided in Table 1. Total scores for this index range from 0 to 100, with a score of 100 demonstrating complete dietary alignment with the DGA [10].

### Non-Energy-Adjusted and Energy-Adjusted Dietary Inflammatory Index

From the total of 45 possible food parameters comprising DII score, a total of 29 were used for calculation as those were the only foods and nutrients available via NHANES data: total energy; carbohydrates; fat: (saturated, monounsaturated and polyunsaturated fatty acids); omega3 and omega6 polyunsaturated fatty acids; protein; grams of alcohol; vitamins A, B1, B2, B6, B12, C, D, E; niacin; fiber; cholesterol; iron; magnesium; zinc; selenium; folic acid; beta carotene and caffeine [7,8]. For the E-DII score, one fewer parameter was used, as the total energy was in the denominator [8]. The scoring components for the DII and E-DII are provided in Table 1. To compute, DII scores were translated into *z*-scores using a global comparative database consisting of data from 11 countries by subtracting the individual’s self-reported value from the mean of the global database then dividing by the standard deviation. These scores were then converted to proportions (i.e. with values ranging from 0 to 1) and centred on zero by doubling each and subtracting 1. These centred proportions were then multiplied by their respective coefficients (overall food parameter-specific inflammatory effect scores) to obtain DII scores for each parameter. These were summed to obtain the overall DII score. E-DII scores were calculated by calculating DII per 1000-kilocalorie consumption and employed the same procedure for scoring but relies on an energy-adjusted global composition database. DII and E-DII scores have a potential range from approximately −9 to +8; i.e. from minimally to maximally pro-inflammatory, respectively. The DII and E-DII are scored similarly and scaled identically, so the scores are comparable across studies [7,8].

### Statistical analysis

Predictor variables included DII and E-DII scores with HEI as the outcome variable. Histograms were used to assess normality of distribution and scatter plots were used to assess the linearity of association of exposure and outcome variables. Pairwise correlations (≤2.5) and variance inflation factor (<0.8) were used to test for collinearity between variables based on previous literature with none detected in the included variables [21,22]. Descriptive statistics were analyzed for the entire sample and by quartiles (Q1–Q4) of DII and E-DII scores. Bivariate analyses were assessed via *t* tests and ANOVA was employed for assessing continuous covariates across quartiles of DII and E-DII scores. Chi^2^ tests were used for categorical covariates across quartiles of scoring. Scheffe *post hoc* analyses were used to test the significance of each possible pairwise comparison in ANOVA analysis.

Linear regression models were used for HEI against DII and E-DII quartiles separately. Unadjusted models were used to assess HEI against DII and E-DII alone. Adjusted models included the covariates of gender, race/ethnicity, education, age, BMI and family income-to-poverty ratios. Covariates were chosen *a priori* as all are associated with dietary intake [12,23–25]. Bootstrap analyses were used to estimate the variance and the 95% confidence interval (CI) for the difference between the coefficients for DII and E-DII from their respective models with HEI as the dependent variable. The difference between the coefficients were determined to be significant if the CI did not include 0 (i.e. *p* < 0.05). As the independent variables (i.e. DII and E-DII) were represented in quartiles, the three coefficients for DII were compared with the corresponding coefficient for E-DII. Each of these coefficients compared one of the quartiles (Q2–Q4) with the referent quartile (i.e. Q1).

All statistical analyses and demographic assessments were survey-weighted. Statistical analyses were conducted using STATA Statistical Software (StataCorp. 2017. Stata Statistical Software: Release 17.0. College Station, TX, USA: StataCorp LLC). *p* Values less than 0.05 were used to assess statistical significance.

## Results

Among this sample of 5829 adults, 51.20% were female, 38.70% were non-Hispanic White, the average age was 50.90 ± 17.40 years and the average BMI was 29.80 ± 0.25 (range = 14.80–84.40 kg/m^2^). The overall average of HEI-2015 scores for the entire sample was 53.12 ± 13.55, the average of DII scores was −0.37 ± 1.79 and the average of E-DII scores was 0.16 ± 1.90. HEI scores were lower (less adherent to the DGA) in quartile 4 of DII and E-DII scores (more pro-inflammatory diet) compared to quartile 1 of DII and E-DII scores (more anti-inflammatory diet) (HEI score in DII quartile 1 = 59.59 ± 0.77; HEI score in DII quartile 4 = 43.48 ± 1.23). Lowest HEI scores were found in quartile 4 of E-DII and highest HEI scores were found in quartile 1 of E-DII (HEI score in E-DII Q1 = 65.68 ± 0.47; HEI score in E-DII Q4 = 40.80 ± 0.54).

Bivariate analyses including Scheffe *post hoc* analyses across quartiles of DII and E-DII are depicted in Tables 2 and 3, respectively. HEI scores, E-DII scores, and all covariates except for age were significantly different across quartiles of DII scores (*p* < 0.001). Additionally, HEI scores, DII scores, and all covariates were significantly different across quartiles of E-DII scores.

**Table 1.** Food groups and nutrients used to assess the HEI-2015, DII and E-DII [7,9].

HEI-2015 components	DII and E-DII
Total fruits^a^	Total energy^c^
Whole fruits^a^	Carbohydrates
Total vegetables^a^	Fat
	Total
	Saturated
	Monounsaturated
	Polyunsaturated
Greens and beans^a^	Fatty acids
	Omega3
	Omega6
Whole grains^a^	Protein
Dairy^a^	Alcohol
Total protein foods^a^	Vitamins A, B1, B2, B6, B12, C, D, E
Dairy^a^	Niacin
Total protein foods^a^	Fiber
Seafood and plant proteins^a^	Cholesterol
Fatty acids^a^	Iron
Refined grains^b^	Magnesium
Sodium^b^	Zinc
Added sugars^b^	Selenium
Saturated fats^b^	Folic acid
	Beta carotene
	Caffeine

^a^These foods are considered “Adequacy” components used to score the HEI-2015.

^b^These foods are considered “Moderation” components used to score the HEI-2015.

^c^This is included only in DII score calculation, not E-DII score calculation.

HEI-2015: Healthy Eating Index-2015; DII: Dietary Inflammatory Index; E-DII: Energy-Adjusted Dietary Inflammatory Index.

**Table 2.** Bivariate analyses by DII quartile among participants from the National Health and Nutrition Examination Survey 2015–2018.

DII quartiles					
*N* = 5289 total sample	Q1: *n* = 1935 (33.2%)	Q2: *n* = 1551 (26.6%)	Q3: *n* = 1259 (21.6%)	Q4: *n* = 1084 (18.6%)	*p* Value
DII quartile range	−4.77 to −1.31	−1.32 to 0.02	0.03–1.38	1.39–4.52	–
Categorical measures (*n* (% per total sample))					
Gender					<0.001*
Males	1176 (20.17%)	760 (13.04%)	519 (8.90%)	388 (6.66%)	
Females	759 (13.02%)	791 (13.57%)	740 (12.70%)	696 (11.94%)	
Race/Ethnicity					<0.001*
Non-Hispanic White	743 (12.75%)	639 (10.96%)	479 (8.22%)	396 (6.79%)	
Hispanic	503 (8.63%)	374 (6.42%)	286 (4.91%)	213 (3.65%)	
Non-Hispanic Black	344 (5.90%)	317 (5.44%)	340 (5.83%)	341 (5.85%)	
Other	345 (5.92%)	221 (3.79%)	154 (2.64%)	134 (2.30%)	
Education					<0.001*
Less than HS	250 (4.29%)	252 (4.32%)	236 (4.05%)	248 (4.25%)	
HS or GED	362 (6.21%)	366 (6.28%)	349 (5.99%)	317 (5.44%)	
Some college	621 (10.65%)	513 (8.80%)	407 (6.98%)	379 (6.50%)	
College or above	702 (12.04%)	420 (7.21%)	267 (4.58%)	140 (2.40%)	
Continuous measures (mean (SD))					
HEI-2015 scores	60.02 (12.98)	53.14 (12.22)	50.03 (11.77)	44.38 (11.67)	<0.001*
E-DII scores	−0.86 (1.91)	0.18 (1.64)	0.69 (1.60)	1.36 (1.53)	<0.001*
Age	50.70 (16.67)	51.02 (17.67)	51.17 (17.65)	51.27 (17.94)	0.8106
BMI	29.29 (6.99)	29.95 (7.19)	30.72 (7.61)	30.78 (7.90)	<0.001*
Family income-to-poverty ratio	2.93 (1.63)	2.60 (1.58)	2.39 (1.52)	2.08 (1.44)	<0.001*

*Significance indicated by a *p* < 0.05.

DII: Dietary Inflammatory Index; Q1–Q4: quartile 1–quartile 4; HS: high school; GED: General Educational Development; HEI-2015: Healthy Eating Index-2015; E-DII: Energy-Adjusted Dietary Inflammatory Index; BMI: body mass index.

**Table 3.** Bivariate analyses by E-DII quartiles among participants from the National Health and Nutrition Examination Survey 2015–2018.

E-DII quartiles					
*N* = 5289 total sample	Q1: *n* = 1500 (25.7%)	Q2: *n* = 1367 (23.5%)	Q3: *n* = 1404 (24.1%)	Q4: *n* = 1558 (26.7%)	*p* Value
E-DII quartile range	−5.8 to −1.09	−1.10 to 0.35	0.36–1.56	1.57–4.36	–
Categorical measures (*n* (% per total sample))					
Gender					<0.001*
Males	589 (10.10%)	685 (11.75%)	730 (12.52%)	839 (14.39%)	
Females	911 (15.63%)	682 (11.70%)	674 (11.56%)	719 (12.33%)	
Race/Ethnicity					<0.001*
Non-Hispanic White	523 (8.97%)	505 (8.66%)	569 (9.76%)	660 (11.32%)	
Hispanic	404 (6.93%)	373 (6.40%)	264 (4.53%)	264 (4.53%)	
Non-Hispanic Black	247 (4.24%)	270 (4.63%)	488 (8.37%)	488 (8.37%)	
Other	326 (5.59%)	219 (3.76%)	146 (2.50%)	146 (2.50%)	
Education					<0.001*
Less than HS	245 (42.16%)	244 (4.19%)	243 (4.17%)	254 (4.36%)	
HS or GED	253 (4.34%)	301 (5.16%)	378 (6.48%)	462 (7.93%)	
Some college	445 (7.64%)	427 (7.33%)	477 (8.18%)	571 (9.80%)	
College or above	557 (9.56%)	395 (6.78%)	306 (5.25%)	271 (4.65%)	
Continuous measures (mean (SD))					
HEI scores	66.65 (10.69)	56.27 (9.41)	49.34 (8.77)	40.75 (8.76)	<0.001*
DII scores	−1.46 (1.51)	−0.60 (1.58)	−0.12 (1.60)	0.65 (1.71)	<0.001*
Age	53.43 (17.56)	52.22 (16.87)	50.21 (17.13)	48.28 (17.50)	<0.001*
BMI	28.88 (6.91)	29.89 (7.06)	30.52 (7.43)	30.89 (7.89)	<0.001*
Family income-to-poverty ratio	2.85 (1.64)	2.65 (1.61)	2.52 (1.57)	2.27 (1.48)	<0.001*

*Significance indicated by a *p* < 0.05.

E-DII: Energy-Adjusted Dietary Inflammatory Index; Q1–Q4: quartile 1–quartile 4; HS: high school; GED: General Educational Development; HEI-2015: Healthy Eating Index-2015; DII: Dietary Inflammatory Index; BMI: body mass index.

There were significantly more males in quartile 1 of DII scores compared to females (*n* = 1176 vs. *n* = 759, *p* < 0.001) while significantly more females were in quartile 4 of DII scores compared to males (*n* = 696 vs. *n* = 388, *p* < 0.001). However, there were significantly more females in quartile 1 of E-DII scores compared to males (*n* = 911 vs. *n* = 589, *p* < 0.001), while significantly more males were in quartile 4 of E-DII compared to females (*n* = 839 vs. *n* = 719, *p* < 0.001). In other words, males had a significantly more anti-inflammatory diet than females according to non-energy adjusted DII scores whereas the same sample of females had a more anti-inflammatory diet compared to males according to E-DII scores.

In both unadjusted DII and E-DII regression models (Table 4), HEI scores were significantly lower (less adherent to DGA) between quartile 2 and quartile 1 of DII scores and quartile 3 and quartile 1 of E-DII scores (HEI and DII Q2 vs. Q1: *β*(SE)= −7.45(0.54), *p* < 0.05; HEI and E-DII Q3 vs. Q1: *β*(SE)= −16.56(0.65) and Q4 vs. Q1: *β*(SE)= −24.93(0.87), *p* < 0.05). HEI scores were lower across E-DII quartiles compared to DII quartiles. Similar results were seen in adjusted models (Table 4). Specifically, there was a consistent increase in beta coefficients across DII and E-DII quartiles in both unadjusted and adjusted models indicating a significant dose-response relationship (DII Q3 vs. Q1: *β*(SE)= −10.68(0.82), *p* = 0.049; E-DII Q2 vs. Q1: *β*(SE)= −9.14(0.64), *p* < 0.05) and Q3 vs. Q1: *β*(SE)= −15.76(0.44), *p* < 0.05 and Q4 vs. Q1: *β*(SE)= −23.77(0.58), *p* < 0.05) (Table 4).

**Table 4.** Multiple Linear regression analyses between HEI, DII and E-DII with and without covariates among 5829 participants from the National Health and Nutrition Examination Survey 2015–2018.

**Unadjusted DII model^a^ (adjusted *R*^2^= 17%)**			
	*β* (SE)	95% Confidence Interval	*p* vs. Referent
DII*			
Quartile 1	Referent	–	–
Quartile 2	−7.45 (0.54)	−14.37 to −0.53	0.046*
Quartile 3	−10.61 (0.85)	−21.46 to 0.25	0.051
Quartile 4	−15.99 (1.76)	−38.35 to 6.37	0.070
**Unadjusted E-DII model^b^ (adjusted *R*^2^= 51%)**			
E-DII**			
Quartile 1	Referent	–	–
Quartile 2	−9.33 (0.77)	−19.17 to 0.51	0.51
Quartile 3	−16.56 (0.65)	−24.85 to −8.26	0.025*
Quartile 4	−24.93 (0.87)	−36.04 to −13.83	0.022*
**Adjusted DII model^c^ (adjusted *R*^2^= 26%)**			
DII*			
Quartile 1	Referent	–	–
Quartile 2	−7.70 (0.74)	−17.08 to 1.70	0.061
Quartile 3	−10.68 (0.82)	−21.10 to −0.26	0.049*
Quartile 4	−16.07 (0.46)	−34.98 to 2.84	0.059
**Adjusted E-DII model^d^ (adjusted *R*^2^= 53%)**			
E-DII**			
Quartile 1	Referent	–	–
Quartile 2	−9.14 (0.64)	−17.30 to −0.97	0.045*
Quartile 3	−15.76 (0.44)	−21.37 to −10.15	0.018*
Quartile 4	−23.77 (0.58)	−31.18 to −16.36	0.016*

*Significance indicated by a *p* < 0.05.

^a^Unadjusted DII model: HEI-2015 and DII by quartile.

^b^Unadjusted E-DII model: HEI-2015 and E-DII by quartile.

^c^Adjusted DII model: HEI-2015, DII by quartile, gender, race/ethnicity, education, age, BMI, family income-to-poverty ratio.

^d^Adjusted E-DII model: HEI-2015, E-DII by quartile, gender, race/ethnicity, education, age, BMI, family income-to-poverty ratio.

DII quartile ranges: 1= −4.77 to −1.31; 2= −1.32 to 0.02; 3 = 0.03–1.38; 4 = 1.39–4.52.

E-DII quartile ranges: 1= −5.80 to −1.09; 2= −1.10 to 0.35; 3 = 0.36–1.56; 4 = 1.57–4.36.

HEI-2015: Healthy Eating Index-2015; DII: Dietary Inflammatory Index; E-DII: Energy-Adjusted Dietary Inflammatory Index.

Results from the bootstrap analyses suggest that the difference between HEI scores for the first versus the fourth quartile of DII scores was significantly larger than the difference between HEI scores for the first versus the fourth quartile of E-DII scores (*ß* = 57.88(2.28); 95% CI: 53.41–62.34; *p* < 0.001). The difference between coefficients for all other quartiles (Q2–Q3) was also significant against the referent Q1 (Q2 vs Q1: *ß*= −54.40(2.28); 95% CI: −58.88 to −49.92; *p* < 0.001; Q3 vs. Q1: *ß*= −12.68; 95% CI: −17.12 to −8.24; *p* < 0.0001).

## Discussion

This cross-sectional secondary data analysis demonstrated that HEI scores were consistently lowest (least adherent to the DGA) across higher DII and E-DII quartiles (more pro-inflammatory diet). HEI scores were lower in the fourth E-DII quartile than in the fourth DII quartile (HEI score: 40.75 ± 8.76 and 44.38 ± 11.67, respectively) and highest in the first E-DII quartile compared to the first DII quartile (HEI score: 66.65 ± 10.69 and 60.02 ± 12.98, respectively). This was further shown to be statistically significant following bootstrap analyses. Results are reflective of the energy-adjustment factor included in E-DII calculations. The E-DII accounted for 52% of the variance in HEI scores in both unadjusted and adjusted models (*R*^2^= 0.52). While only 17% of the variance in HEI score is explained by the DII in the unadjusted model (*R*^2^= 0.17), this increased to 26% of the variance in HEI scores explained by the DII in the adjusted model (*R*^2^=0.26). Thus, E-DII scores may explain variability in HEI scores better than DII scores among this sample of adults. This is not unexpected as both the HEI and E-DII account for energy intake in their calculations whereas the DII does not.

Previous studies assessing the DII with the HEI have yielded similar results as this analysis. For example, DII scores were found to significantly increase as HEI-2010 scores decreased in a sample of American young adults [14]. In other samples of American adults, the DII and E-DII has been positively associated with chronic diseases (i.e. rheumatoid arthritis, cancer), while the HEI is negatively associated with chronic diseases among the same samples of adults [15,26]. While this inverse relationship between the inflammatory indices and the HEI is similar to the findings in this secondary data analysis, this analysis differs in that the DII and E-DII are being analyzed in one analysis against a national metric to assess the effectiveness of the 1000 kilocalorie adjustment when assessing the inflammatory potential of this diet.

Demographics across DII and E-DII quartiles were similar except for age and gender. It should be noted that the average BMI for the total sample of adults used in this analysis was 29.80 kg/m^2^±0.25, indicative of an average overweight BMI for this sample. Additionally, when broken down by DII and E-DII quartiles, average BMI was consistently overweight or obese. Age was not significantly associated with DII scores but was significantly associated with E-DII. Specifically, a higher average age was observed in the first quartile of E-DII compared to all other quartiles. This difference suggests that energy adjustment is important when assessing inflammatory potential of the diet against age. According to previous literature among healthy American adults, appetite and energy intake are lower in older adults than in younger adults [27]. Therefore, adjusting for energy is important to account for these changes with increasing age. The inverse association between DII score and energy intake is also consistent with what was reported in other studies. As such, this is what prompted development of the E-DII [28–30].

With regard to gender, there was a greater abundance of males in the first quartile of DII scores and females in the first quartile of E-DII scores. Conversely, there were a greater number of females in the fourth quartile of DII scores and males in the fourth quartile of E-DII scores. Therefore, when looking at this sample of adults, males had more anti-inflammatory diets than females according to the DII whereas females had more anti-inflammatory diets than males according to the E-DII. It should be noted that differences have been found previously between males and females such that males tend to have higher energy intake compared to females [31,32]. Nutrient intake is correlated with energy intake, such that nutrient intake increases as caloric intake increases as more food is being consumed [33,34]. Therefore, an increase in caloric intake can impact unadjusted DII scores. Without energy adjustment, those who consumed more calories may have greater consumption of both anti- or pro-inflammatory nutrients, which can impact DII scores. With the 1000-kilocalorie adjustment, however, the influence of energy intake is mitigated as nutrient intake is being standardized across all participants. Similarly, this was observed in other studies and is consistent with the inverse energy intake gradient with DII scoring [28–30]. Consequently, this discrepancy between male and female energy intake likely explained the difference in distribution of gender between quartiles of DII and E-DII. Additionally, there were significant associations between quartiles of both DII and E-DII among race/ethnicity, education, BMI, and family income-to-poverty ratio; nevertheless, this was expected as it is noted that these variables impact dietary intake [12,23–25].

Overall, results suggest that the E-DII regression model explained more of the variability observed in HEI scores compared to the DII; furthermore, as both the HEI and E-DII are assessed on a per 1000 kilocalorie basis, E-DII may be a more appropriate counterpart for assessment alongside of the HEI than the non-adjusted DII. In support of concurrent or tandem use of these dietary assessment scores, it should be noted that assessing individual nutrients alone as in the E-DII can result in incomplete dietary assessment as foods are made up of many nutrients that exert their synergistic effects in the body rather than in isolation [35]. Therefore, when assessing nutrients individually, there is a potential for attributing an effect to one nutrient rather than the multiplicity of nutrients comprising a specific food. Nevertheless, assessing E-DII along with HEI may provide further insight into dietary quality and its potential to influence inflammation and subsequent chronic disease. This is especially true as assessment of HEI solely against an individual’s inflammatory status has yielded null findings in previous publications [1,36]. For example, investigations have found a lack of association between HEI scores and inflammatory biomarkers such as CRP, IL-6, and TNF-α [1,36]. While HEI remains a validated metric of diet quality with inverse associations with chronic disease [37], the null associations between HEI scores and inflammatory biomarkers in previous analyses leaves the E-DII as a valuable accompaniment to the HEI when assessing dietary-induced inflammation. Taken collectively, assessing dietary quality using both food groups (HEI) and individual nutrients (E-DII, which also included some whole foods) may improve the assessment of interrelationships among nutrients that comprise whole food groups and subsequent health outcomes especially inflammatory-based chronic diseases [33,35].

Strengths of this analysis include the use of NHANES data derived from a nationally representative sample of US adults. Additionally, this is the first study to assess the HEI against both the DII and E-DII in the same population. Nevertheless, this study is not without limitations. The use of two 24-hour recalls may not reflect usual dietary intake for participants. Further, 24-hour recalls are prone to reporting biases associated with self-report, despite the fact that NHANES researchers are extensively trained to reduce such bias. Another limitation is that not all nutrients used to calculate DII and E-DII were available in the NHANES database. While this is a common limitation in the application and use of these calculations, it may make comparisons between the literature difficult as studies are not always consistent in the nutrients assessed [38–40]. However, validation studies indicate the predicative ability of the DII on inflammatory markers is not diminished when as few as 19 components are included compared with a full set of DII components [41,42]. Nevertheless, as the number of food components used in this analysis is similar to previous publications, the results of this study allow for cross-study comparison [43–48]. Finally, these findings are not generalizable to other age groups or populations beyond this assessment.

## Conclusions

While the DII is widely used to assess the inflammatory potential of the diet, its lack of an energy adjustment is of concern. Therefore, the E-DII was developed to assess inflammation from the diet on a 1000-kilocalorie basis. This energy adjustment not only ensures non-inflated results when assessing the inflammatory potential of the diet but also provides a more comparable metric to the HEI, which is also adjusted per 1000 kilocalories. This analysis found E-DII to be significantly associated with HEI scores and explained more variation in HEI scores than DII scores. Therefore, the E-DII may serve as a concomitant metric with HEI when assessing diet’s association with inflammation with the HEI. As the HEI remains a validated method of assessing dietary quality, its use along with the E-DII allows for a greater understanding of the role of food groups and nutrients on the diet–inflammation relationship.

## Data Availability

Data sharing is not applicable to this article as no new data were created or analyzed in this study.
